# Book Reviews

**Published:** 1910-11

**Authors:** 


### BOOK REVIEWS.

A TEXT-BOOK OF PHARMACOLOGY AND THERAPEUTICS; OR THE ACTION OF DRUGS IN HEALTH AND DISEASE. By Arthur R. Cushny, M. A., M. D., F. R. S., Professor of Pharmacology in the University of London, Manchester, Oxford and Leeds; formerly Professor of Materia Medica and Therapeutics in the University of Michigan. Octova, 74 pages, with 61 engravings. Cloth· $3.75 net. Lea & Febiger, Publishers, Philadelphia and New York, 1910.

The original appearance of this book may fairly be said to have marked a new departure, namely, the general acceptance of the fact that the real action of drugs is knowable, and that therapeutics is a rational science even though much ground still occupied by empiricism remains to be conquered. There is no doubt that this volume had much influence in hastening this new era, and in stimulating its advancement. The interest evoked in the subject is seen in its five large editions demanded within eleven years. It has become a text-book in leading medical colleges, and is sought by the practitioner who desires to understand the *modus operandi* of drugs so that he may prescribe intelligently and efficaciously.

---

A MANUAL OF OBSTETRICS. By A. F. A. King, M. D., Professor of Obstetrics and Diseases of Women in the Medical Department of the George Washington University, Washington, D. C., and in the Medical Department of the University of Vermont, etc. Eleventh edition, enlarged and thoroughly revised, 12mo., 713 pages with 341 illustrations and three colored plates. Cloth, $2.75, net. Lea and Febiger, Philadelphia and New York.

The original aim of the author has in this revision been judiciously maintained. Brevity and lucidity of statement mark its pages without sacrifice of completeness, and as a text-book for the elementary student or work of reference for the overbusy practitioner, it will continue to enjoy its well merited reputation.

Among the more important additions may be mentioned the subjects of pubiotomy, spontaneous version by posture and the factor of thigh-pressure upon the abdomen considered as one of the auxiliary forces of labor. The section on hyperemesis has been rewritten and the illustrations have been increased by the addition of thirty-seven new figures.

---

PATHOGENIC MICRO-ORGANISMS, INCLUDING BACTERIA AN DPROTOZOA. A Practical Manual for Students, Physicians and Health Officers. By William H. Park, M. D., Professor of Bacteriology and Hygiene in the University and Bellevue Medical College, and Director of the Research Laboratory, Department of Health, New York City; and Anna W. Williams, M. D., Assistant Director of the Research Laboratory. New (fourth) edition, thoroughly revised. Octavo, 670 pages, with 196 illustrations and 8 full-page plates. Cloth, $3.75 net. Lea & Febiger, Publishers, Philadephia and New York, 1910.

Among the host of works on bacteriology, Professor Park's book won a foremost place immediately on its original appearance, and in the intervening eleven years four large editions have been required. Its firm establishment as as tandard and favorite is thus beyond question. In its previous edition the author broadened it to include the protozoa as well as the bacteria, the animal as well as the vegetable germs, two parts of the great subject of pathognic microorganisms, so that this single volume presents the entire field in the most convenient and authoritative manner. In this new edition the scope of the book has been broadened again by including microorganisms important in agriculture.

---

THE PRINCIPLES OF PATHOLOGY. VOLUME 1, GENERAL PATHOLOGY. By J. George, Adami, M. A., M. D., LL. D., F. R. S., Professor of Pathology in McGill University, Montreal. New (2d) edition, thoroughly revised. Octavo, 1027 pages, with 329 engravings and 18 plates. Cloth, $6.00 net. Lea & Febiger, Publishers, Philadelphia and New York, 1910.

The rapid absorption of the very large first edition testifies both t othe value of this work and to the discriminating judgment of the profession. Pathology has come to be recognized in its rightful place as fundamental to all practice, and the furthe1 fact is now appreciated that the path of least resistance in mastering a subject is to grasp its principles and group around them the knowledge of details. Professor Adami's book, coming from the pen of one of the world's foremost pathologists, presents the whole field of general pathology with the highest authority, and in conformity with the aforseaid rational method of comprehension. He is master of a clear and charming literary style, hence his pages rivet the attention and memory. His book appeals to a wide circle of readers, for it has become a text in colleges of the best class, and it interests every practitioner who wishes to understand the ultimate nature of disease. Short as the interval has been since original publication, Professor Adami has made extensive changes in his book, rearranging it, rewriting sections, and incorporating the latest developments in its most progressive department. This is exemplified by the revision of every topic to date, and by the inclusion of much entirely new matter, for instance the section on the ultramicroscopic causes of disease, a subject which has come into prominence while the volue was passing through press.

---

A TREATISE ON ORTHOPEDIC SURGERY. By Royal Whitman, M. D., Adjunct Professor of Orthopedic Surgery in the College of Physicians and Surgeons, New York; Professor of Orthopedic Surgery in the New York Polyclinic. New (4th) edition, revised and enlarged. Octavo, 908 pages, with 601 illustrations, mostly original. Cloth, $5.50, net. Lea & Fibiger, Philadelphia and New York, 1910.

The new edition of Whitman's Orthopedic Surgery, will establish even more firmly the high position won by its three predecessors. The opportunity to prevent lifelong deformity by correction applied when the body is young and plastic brings this subject within the duties of the family practitioner, for he it is who first sees such defects. They form a large share of general surgery, hence such a work interests every surgeon, as well as the specialist. Dr. Whitman has improved every opportunity to revise his sterling work, and this new issue is no exception. The changes have been so extensive that the entire book has been re-set.

